# Draft Genome Sequence of the Anaerobic Ammonium-Oxidizing Bacterium “*Candidatus* Brocadia sp. 40”

**DOI:** 10.1128/genomeA.01377-16

**Published:** 2016-12-08

**Authors:** Muhammad Ali, Mohamed Fauzi Haroon, Yuko Narita, Lei Zhang, Dario Rangel Shaw, Satoshi Okabe, Pascal E. Saikaly

**Affiliations:** a Water Desalination and Reuse Center (WDRC), Biological and Environmental Science and Engineering (BESE) Divison, King Abdullah University of Science and Technology (KAUST), Thuwal, Saudi Arabia; b Department of Organismic and Evolutionary Biology, Harvard University, Cambridge, Massachusetts, USA; c Division of Environmental Engineering, Faculty of Engineering, Hokkaido University, Sapporo, Hokkaido, Japan

## Abstract

The anaerobic ammonium-oxidizing (anammox) bacterium “*Candidatus* Brocadia sp. 40” demonstrated the fastest growth rate compared to others in this taxon. Here, we report the 2.93-Mb draft genome sequence of this bacterium, which has 2,565 gene-coding regions, 41 tRNAs, and a single *rrn* operon.

## GENOME ANNOUNCEMENT

Anaerobic ammonium-oxidizing (anammox) bacteria have the ability to convert NH_4_^+^ to dinitrogen gas using NO_2_^−^ as an electron acceptor and were first discovered in a bench-scale nitrifying reactor (1). Anammox bacteria were characterized as chemolithoautotrophs and are affiliated with the phylum *Planctomycetes* (2). Five candidate genera, “*Candidatus* Kuenenia,” “*Ca.* Brocadia,” “*Ca.* Anammoxoglobus,” “*Ca.* Jettenia,” and “*Ca.* Scalindua,” have been proposed in this bacterial lineage to date. Although no pure culture is currently available, several monospecies enrichment cultures have been obtained for anammox bacteria. Anammox-based technologies are widely applied for the treatment of NH_4_^+^-rich wastewater (3). However, the application window of this energy- and resource-efficient process is restricted due to the low growth rate of anammox bacteria (4, 5). Recently, “*Ca.* Brocadia sp. 40” demonstrated a maximum specific growth rate (*μ*_max_) of 0.33 days^−1^ (6), which is four times higher than the highest value reported so far for anammox bacteria. Here, we describe a high-quality “*Ca.* Brocadia sp. 40” genome recovered from shotgun metagenomic sequencing of an enrichment culture.

Initially, an anammox bacterium affiliated with “*Ca.* Brocadia sp. 40” was enriched from brewery wastewater sludge in a fixed-bed anammox reactor (7). Subsequently, “*Ca.* Brocadia sp. 40” cells were further enriched in a membrane bioreactor. Genomic DNA was extracted using a DNA extraction kit (FastDNA SPIN kit for soil, MP Biomedicals). The library was prepared using a TruSeq Nano DNA library preparation kit (Illumina) and sequenced using a HiSeq 4000 platform (Illumina). Reads were quality-trimmed using Trimmomatic version 0.35 (8) and assembled using SPAdes version 3.9.0 (9). The “*Ca.* Brocadia sp. 40” genome was binned based on coverage and genomic properties using MetaBAT version 0.30.3 (10) as previously described (11, 12).

A 2.93-Mb genome sequence comprising 122 contigs (42.7% GC content) was obtained, and 2,565 gene-coding regions, 41 tRNAs, and a single *rrn* operon were annotated. The genome is 93% complete based on an analysis with CheckM version 1.0.5 (13). The genome size of “*Ca.* Brocadia sp. 40” is smaller and encodes less genes than other anammox species. The nearest evolutionary neighbor, “*Ca.* Brocadia fulgida” (96% 16S rRNA gene sequence similarity), has a genome size of 3.55 Mb (14). This suggests that a smaller genome size is potentially associated with the higher growth of “*Ca*. Brocadia sp. 40” as reported elsewhere (15). The “*Ca.* Brocadia sp. 40” genome encodes gene clusters for core anammox metabolism. However, similar to “*Ca.* Brocadia sinica” (16), conventional nitrite reductase genes (*nirS* and *nirK*) were missing in the “*Ca.* Brocadia sp. 40” genome. It was concluded that anammox bacteria related to the “*Ca.* Brocadia” genus employ an unknown nitrite reductase, which should be further investigated.

### Accession number(s).

The “*Ca.* Brocadia sp. 40” draft genome was deposited at DDBJ/ENA/GenBank under the accession number MJUW00000000.
